# Early death in Munchausen syndrome: A case report

**DOI:** 10.1002/ccr3.2254

**Published:** 2019-06-20

**Authors:** Rosaria Di Lorenzo, Ludovica Lannocca, Maritea Burattini, Andrea Vasta, Martina Galletti, Alessandro Minarini, Francesca Mongelli, Salvatore Sportiello, Sergio Rovesti, Paola Ferri

**Affiliations:** ^1^ Department of Mental Health and Drug Abuse, Psychiatric Intensive Treatment Facility AUSL Modena Modena Italy; ^2^ School of Nursing University of Modena and Reggio Emilia Modena Italy; ^3^ Department of Mental Health and Drug Abuse AUSL Modena Modena Italy; ^4^ Department of Biomedical, Metabolic and Neural Sciences University of Modena and Reggio Emilia Modena Italy; ^5^ ASL Napoli 2 Nord U.O.S.M. Afragola Afragola, Napoli Italy

**Keywords:** factitious disorder, factitious physical and psychological symptoms, gridiron abdomen, Munchausen syndrome, wandering

## Abstract

This case contributes to raising awareness and understanding of the complex clinical presentations of Munchausen syndrome (MS). Education of staff to the seriousness and genuineness of this disorder should be implemented, especially in hospital units, in order to detect such complex clinical situations quickly and accurately, preventing iatrogenic risks.

## 1 INTRODUCTION

Munchausen syndrome (MS) was first described in 1951 by Asher, who reported individuals who intentionally produced signs and symptoms of a disorder to seek medical care.^1^ The author defined this as the “Syndrome of Munchausen” due to similarities with the fantastic stories of Baron Karl Friedrich Hieronymus Freiherr von Münchausen (1720‐1797). The use of this name has, however, been strongly criticized and, over the years, several names have been proposed: hospital addiction syndrome, patients with wandering problems, or even hospital vagabonds, etc.^2^ MS is included in the Tenth Edition of the International Classification of Diseases and in the Fifth Edition of the Diagnostic and Statistical Manual of Mental Disorders as “factitious disorder.”^3^, ^4^

A recent review estimated the prevalence of factitious disorders ranged between 0.6% and 3% in general medicine and psychiatry settings and between 0.02% and 0.9% in other specialist clinics.^5^ This disorder is more often reported among men than women and frequently occurs in early adulthood. Possible predisposing factors are represented by long‐term treatments or hospitalizations in childhood or adolescence resulting from medical conditions, the presence of grievances against medical professionals, work experience in health care and personality disorders.^6^

Folks and Freeman described three features of MS: (a) fake recurrent illnesses; (b) wandering; and (c) fantastic pseudology, described as a sort of pathological lie aimed at self‐gratification which represents a symptom ranged between conscious deceit and delusion, without the patient losing touch with reality.^7^, ^8^

Individuals with MS complain predominantly of physical symptoms and often abuse analgesics, sedatives, and other drugs to produce symptoms that suggest mental disorders.^9^ The repetitive hospitalizations and invasive medical and surgical procedures experienced by patients with MS often lead to iatrogenic consequences.^10^ Physical signs, which testify to these unnecessary procedures, can be illustrated by the so‐called “gridiron abdomen,” a condition characterized by multiple abdominal scars resulting from invasive surgeries.^11^, ^12^ According to some authors, factitious disorders represent an elaborate modality for avoiding painful emotions by shifting all attention on the body in order to camouflage emotional trauma, which is difficult to elaborate.^13^

If patients with MS are recognized by physicians or other health professionals and are confronted about the fraudulent nature of their symptoms, they often deny and leave the hospital without formal discharge.^14^ Later, they look for another hospital or health facility for new admissions.^15^, ^16^ For this reason, epidemiological studies and clinical trials to establish therapeutic strategies for MS are difficult to conduct because patients deny suffering from a psychiatric disorder and lack cooperation regarding treatment.^10^, ^11^, ^17^, ^18^

In this report, we describe the case of a Caucasian male patient in his 20s, with a clinical history of repetitive hospital admissions due to factitious symptoms.

## 2 CASE PRESENTATION

### 2.1 The initial encounter with the patient

We met the patient for the first time in the Emergency Room (ER) of a general hospital where he requested a psychiatric consultation due to suicidal ideation following a bereavement. He justified this request with a recent history of anxiety and conflict related to his suicidal impulse, which had led him to interrupt his train journey he was making from an Italian town to his city of residence. He was in his 20s, had an unkempt appearance, and was clearly overweight. At the first evaluation carried out in the ER, he appeared lucid, oriented both in time and space, with normal vital signs. Old and recent extensive scars on his abdomen and thorax were noted, likely due to multiple exploratory laparotomies and surgeries. He was carrying the medical documentation of a 3‐day stay in the psychiatric unit of a hospital in another town, where he had been admitted for a massive ingestion of drugs and from which he had been discharged that same morning with the diagnosis of “Factitious disorder with prevailing physical symptoms.” This medical documentation also reported hypothyroidism, hypogonadism, and arterial hypertension as physical symptoms. The patient spontaneously told us that, before his last hospitalization, he had been treated in a rehabilitative community for a few months. Moreover, he informed us that during his last hospitalization, a surgical consultation had been performed to remove sutures applied following an exploratory laparotomy which he had undergone for suspected appendicitis in a hospital in central Italy. During this first psychiatric assessment in our ER, we also noticed he had a long‐term habit of wandering which he began at the age of majority. He described difficult relationships with his family members and reported that since the age of eighteen he had frequented various health facilities in different places.

The medical history offered by the patient revealed numerous hospitalizations for a large variety of surgical, internist, and psychiatric disorders, in many Italian cities and even abroad. The patient provided only partial and vague information about the motivations for his previous hospitalizations and informed us that he had to take a lot of drugs due to many pathologies. Among the drugs were ramipril 5 mg and amlodipine 5 mg for hypertension and testosterone undecanoate 80 mg because of the removal of both testicles, which had occurred a few years earlier during one of his multiple operations. He was not able to give us clear motivations for this mutilating operation.

After this first consultation, the patient underwent laboratory tests, which did not show any acute organic pathology. After a period of observation in the ER, he was transferred to the psychiatric unit of our hospital. During the initial days of hospitalization, several interviews were conducted with the patient, in which he appeared frankly reticent and elusive in his answers. Finally, he revealed that he was being treated in a psychiatric community health service in the city of his residence.

In order to identify the patient's history in detail, we telephoned the patient's psychiatrist at the outpatient community health service of his town. His psychiatrist confirmed our suspected diagnosis of MS, reporting that the patient had had many hospital admissions due to a variety of factitious disorders and frequent requests for ER consultations for various and continuously changing physical symptoms in many Italian cities. The patient had also undergone many exploratory surgical procedures, always with negative results. His total lack of awareness of suffering from a psychiatric illness and his compulsive and endless wandering did not permit regular psychiatric treatment. We collected his family history: The father had died when the patient was still a child and his mother had later entrusted him to an institute for minors due to her work commitments, which would not allow her to take care of him. It was during his stay in this institute that he showed the first factitious symptoms, requesting medical consultation. The patient had an older brother who lived on his own in the patient's city of residence, but there was no contact between the two.

During the hospitalization, the ward physicians phoned his mother to inform her of her son's hospitalization and, at the same time, to collect additional information about his history. On that occasion, they could appreciate that she was not worried about her son, showing a detached attitude toward him. She said she was exasperated by his continuous wandering (“because he will never change…”) and asked not to be involved in his problems anymore.

During the hospitalization, the patient appeared to be collaborative, calm, and adherent to the prescribed therapies (quetiapine 600 mg/d, mirtazapine 30 mg/d, and zolpidem 10 mg/d), but openly contested being sent home to continue psychiatric treatment with his psychiatrist. After 2 days of hospitalization, he was discharged with instructions to continue treatment with his psychiatrist, who was in turn informed of his patient's discharge.

### 2.2 The subsequent encounters with the patient

After about 2 years, the patient asked for a new consultation at our ER of our hospital, complaining of major distress and intentions of self‐harm, with the same vagueness and elusiveness as at his previous consultation. He spontaneously requested a new psychiatric admission. The patient confessed that a few days before, he had been admitted to the urological unit, after reporting severe scrotal pain despite the fact that his testicles had been removed years before. There he had undergone exploratory surgery and was promptly discharged without any therapy. After psychiatric consultation, he was again hospitalized in the psychiatric ward, where the ward psychiatrists collecting his medical history learned that, in the same year, he had undergone another exploratory laparotomy in the hospital of another town because of severe pelvic pain and was immediately transferred to the psychiatric service of that same hospital. Subsequently, after many hospitalizations in different units of other cities for brief periods, he remained at home for few months, during which time he was regularly examined by his mental health physician and showed normal behavior. After a week of hospitalization in our acute psychiatric ward, he was transferred to the psychiatric ward of his hometown, in order to continue the therapeutic and rehabilitative program at the psychiatric service of territorial jurisdiction. Colleagues were also advised of his wandering behavior aimed at obtaining unnecessary and dangerous surgical interventions.

During the subsequent year, the patient was admitted to the cardiology ward of our hospital, due to a suspected cardiomyopathy of vascular origin, for which several investigations had been planned, including a coronary angiography and a transesophageal ultrasound scan. Before proceeding, the cardiologists, given the bizarre behavior exhibited by the patient and the incoherence of symptoms reported, decided to request a psychiatric consultation. When the patient met the psychiatrists who had followed him in the previous psychiatric hospitalization in our town, he promptly self‐discharged from the hospital.

### 2.3 Subsequent history reported by the patient's psychiatrist

After the above‐referenced self‐discharge from the cardiology unit of our general hospital, the patient was admitted to the intensive observation ward in another hospital for suspected aortic dissection. Examination results were negative and the patient was discharged. Later that month he was transported by helicopter to yet another city for suspected acute heart disease, despite the previous investigations performed.

In August of the same year, he returned home and remained there until September, when he resumed his wandering from one hospital to another in different Italian cities.

In the following year, the patient, after undergoing yet another exploratory urological procedure, was admitted within a few days to the hospitals of three Italian towns, distant from each other, due to chest pain, phlebitis, pelvic pain. By the end of the same year, the patient had been hospitalized for brief periods at the hospitals of many northern, central, and southern Italian towns. Afterward, he managed to stay at home for a year, when he was again admitted to a hospital to undergo a full body computerized axial tomography scan, and subsequently, he was twice hospitalized in two Italian towns.

**Figure 1:**
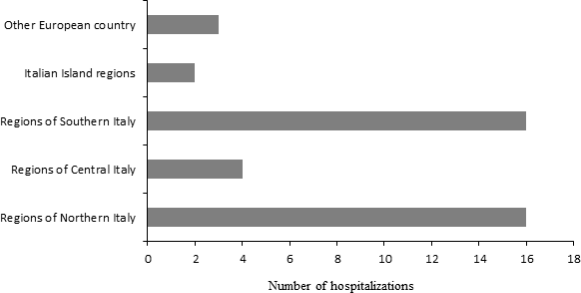
Number and place of hospitalizations in a 4‐year period

The following year, he was hospitalized abroad, in the capital city of another European country, where, in September, he was discharged and wandered for about 2 months. When he finally returned to Italy, he stayed at home until December, when he resumed his wanderings, going to hospitals of different towns, where he underwent new exploratory interventions for pelvic pains. Between February and May of the following year, the patient visited many hospitals in ten different cities, moving through Italy, from north to south, including the islands. Finally, he collected 41 hospitalizations both in Italy and another European country in a period of 4 years (Figure 1). After that, he stayed at home for over a year until October of the following year, when he was voluntarily admitted to a local psychiatric ward in the hospital of his town, and where, after having peripheral edema in the lower limbs, he suddenly died of suspected acute heart failure.

## 3 DISCUSSION

This case can be considered emblematic of other MS cases reported in the literature: The patient's somatic symptoms were vague, diffuse, nonspecific, and, after in‐depth examination, seemed to be controlled by the patient himself.^11^ Also, the patient's recollection was vague and inaccurate; his body revealed multiple signs of exploratory (and sometimes mutilating) surgeries. He carried a rich medical record where no clear clinical diagnosis was given for his various complaints. His approach was characterized by manipulations and lies, and his behavior was marked by frequent flights from hospitals especially when he was “discovered.” Also, his family and childhood history were characterized by the same features described in many cases in the literature: isolation, lack of adequate family support, and early institutionalization.^11^ In addition, this case presented other characteristics of factitious disorder not immediately recognized: He did not have any relatives to interview, there was no primary physician responsible for his care registered on his documents, and he was socially isolated.^15^ Although these elements could have suggested the factitious nature of his symptoms and signs, he underwent many medical treatments because his physical complaints were frequently convincing.^5^ When the patient requested psychiatric consultation, he complained of psychiatric symptoms simulated in order to be hospitalized even if in a psychiatric ward. His wandering behavior from one hospitalization to another, started early, when he was 18, and was always aimed toward being hospitalized again in a compulsive and stereotyped way, representing a chronic “hospital peregrinating” without managing any appropriate treatment plan, as previously described in literature.^19^ Many explanations can be given for his wandering behavior: a compulsive need for attention and assistance by healthcare professionals; looking for a family; institutional dependence which began in his childhood; a regressive need to be cared for as a child, etc. Distinct from several other psychiatric disorders, the factitious symptoms presented by our patient were not characterized by an altered perception of reality (such as delusion) nor by an altered state of consciousness (such as delirium) nor by a pathological conviction of illness (such as hypochondria), but by the compulsive need to undergo medical treatment and/or surgical invasive procedures, often in inpatient hospital settings.^11^

Moreover, our patient, as all individuals with factitious disorder, developed health problems and symptoms connected to his self‐harm behavior involving frequent and harmful invasive procedures. The most severe iatrogenic complication in our case was the mutilation of both testicles likely due to pelvic pain complaints. On the contrary, the lack of necessary examination and treatments, caused by his inability to follow a proper therapeutic plan, probably contributed to his early death, due to a cardiac complication. As in a previously described case,^20^ also in this case we believe a genuine organic illness probably coexisted with mimicked physical disorders, but our patient, due to the psychiatric disorder, did not permit us to correctly evaluate and treat it.

## 4 CONCLUSIONS

The clinical course of this case was severe and was characterized by a pattern of repetitive wandering from one hospital to another and a firm refusal of appropriate examinations and therapies that likely contributed to his early death. The clinical history of our patient can serve to illustrate to physicians the need to better diagnose MS in order to provide appropriate treatments and avoid invasive procedures and mutilating operations. The early death of our patient reminds us of the risk of factitious symptoms in hampering the correct diagnosis and treatment of real, potentially deadly, disorders. The availability of electronic files with patients’ health information as well as a good cooperation between inpatient and outpatient services could help health professionals to diagnose these complex cases early, avoiding invasive procedures or repetitive hospitalizations. Education of staff to the seriousness and genuineness of this disorder should be implemented especially in hospital units. For successful treatment, an early recognition and accurate diagnosis are necessary, followed by the maintenance of patients in a regular long‐term treatment setting, where supportive, and later, confrontational, psychotherapy can be conducted in order to foster more appropriate illness awareness and treatment adherence by patients. Finally, in light of our case, we would like to indicate that a multi‐professional staff could permit a more appropriate approach to patients with MS, avoiding the risk of both excessive and missing diagnostic and treatment procedures. More research is needed in order to understand the cultural, social, and psychological characteristics of this disorder and to discover beneficial preventive and treatment strategies for reducing the extraordinary wasted costs and efforts to patients affected by the disorder and to the physicians and all healthcare workers who care for them.

## CONFLICT OF INTEREST

None declared.

## AUTHOR CONTRIBUTIONS

RDL had a major role in the conception of the study, in reporting clinical information and in writing the manuscript; LL contributed to the study and made a detailed literature collection on this topic; MB, AV, and SS reported important clinical information on this case; MG, AM, and FM participated in collecting clinical information and writing the manuscript; SR and PF supervised the design of the study and revised the manuscript. All Authors made substantial contributions to conception and design of this case report and were involved in drafting the manuscript or revising it critically for important intellectual content. All Authors read and approved the final manuscript.
